# Effects of Temperature and Water Vapor Content on Microstructure, Mechanical Properties and Corrosion Behavior of C/C-SiC Composites

**DOI:** 10.3390/ma17246259

**Published:** 2024-12-21

**Authors:** Yanbin Wei, Zhiyong Ye, Yalei Wang, Xiang Xiong, Zaidong Liu, Jinming Wang, Tongqi Li

**Affiliations:** 1 National Key Laboratory of Science and Technology on High-Strength Structural Materials, Central South University, Changsha 410083, China; ybweicsu@163.com (Y.W.); pmri_yezhiyong@csu.edu.cn (Z.Y.); xiongx@csu.edu.cn (X.X.); zaidongliu@csu.edu.com (Z.L.); 2 Aerospace Research Institute of Materials and Processing Technology, Beijing 100076, China; wangjinming@gmail.com (J.W.); tongqi_l@126.com (T.L.)

**Keywords:** C/C-SiC composites, water vapor corrosion, microstructure, flexural properties

## Abstract

Carbon-fiber-reinforced carbon and silicon carbide (C/C-SiC) composites were prepared using chemical vapor infiltration (CVI) combined with reactive melt infiltration (RMI). The microstructure and flexural properties of C/C-SiC composites after oxidation in different temperature water vapor environments were studied. The results indicate that the difficulty of oxidation in water vapor can be ranked from easy to difficult in the following order: carbon fiber (CF), pyrolytic carbon (PyC), and ceramic phase. The surface CFs become cone-shaped under corrosion. PyC has a slower oxidation rate and lower degree of oxidation compared to CF. The SiO_2_ layer formed by the oxidation of SiC and residual Si was insufficient to fully cover the surface of CFs and PyC. As the temperature increased, the oxide film thickened, but the corrosion degree of CF and PyC intensified, and the flexural performance continuously deteriorated. The flexural strength of C/C-SiC composites was 271.86 MPa at room temperature. Their strength retention rates were all higher than 92.19% after water vapor corrosion at 1000 °C, still maintaining the “pseudoplastic” fracture characteristics. After water vapor corrosion at 1200 °C, the CFs inside the composites sustained more severe damage, with a strength retention rate as low as 48.75%. The fracture mode was also more inclined towards brittle fracture.

## 1. Introduction

To improve the payload capacity of rockets, advancements are being made in the upper-stage liquid rocket engine to achieve higher thrust, increased specific impulse, and a lower weight [1,2,3]. The nozzle plays a critical role in the thrust chamber of a rocket engine. It functions as the exhaust for high-temperature and high-pressure gases from the combustion chamber, exerting a direct impact on the engine’s performance [4]. C/C-SiC composites, known for their exceptional heat resistance, mechanical strength, fatigue resistance, oxidation resistance, corrosion resistance, and low density, have become the preferred material for the extension section of upper-stage engine nozzles [5,6,7]. In contrast to conventional metal nozzle extension sections, the density of C/C-SiC composites does not exceed 2.5 g/cm^3^, which is only 25% to 33% of the density of metal nozzle materials. In addition, these composites can achieve a working temperature of 1600 °C, significantly surpassing the temperature resistance of traditional metal materials [8]. In contrast to the extension section of C/C composite nozzles, C/C-SiC composites demonstrate a notable level of resistance to oxidation at elevated temperatures, thereby facilitating extended operational durations.

The use of C/C-SiC composites in the nozzle of upper-stage rocket engines requires addressing challenges associated with material oxidation and corrosion problems caused by the gas. The gaseous environment of engines varies greatly due to the use of different propellants. However, the primary gas components comprise oxidizing atmospheres, including oxygen and water vapor. It is necessary to investigate the oxidation and corrosion behavior of C/C-SiC composites in atmospheres containing oxygen and water vapor. The oxidation behavior of C/SiC composites in oxygen or air has been investigated by several researchers [9,10,11,12,13]. They emphasized the protective effect of SiO_2_ on the internal structure during the antioxidant process [14,15], but provided limited information on the oxidation behavior of CF and carbon matrix. Nevertheless, for C/C-SiC composites with low SiC content, the SiO_2_ layer formed by oxidation in air is difficult to isolate from the air [13,16,17]. Additionally, water vapor can impair the SiO_2_ oxide layer, especially when the water vapor content is high [13]. E.J. Opila [18] highlighted the substantial impact of water vapor on the oxidation behavior of SiC materials. The presence of water vapor not only elevates the oxidation rate of SiC but also disrupts the oxide film of SiO_2_, leading to the appearance of a loose and porous SiO_2_ on the surface. The corrosion of the surface SiO_2_ protective film by water vapor accelerates the oxidation of the PyC interface phase and CFs, resulting in a significant performance decline of the composites. Water vapor still plays an important role in the oxidation process of C/SiC composites, and the partial pressure of water vapor directly influences the oxidation rate of the composites. The water vapor selectively reacts with carbon in the composites, leading to a reduction of the available SiC fraction for reaction with water vapor [19,20]. Therefore, the oxidation of the carbon phase is a crucial aspect of the overall oxidation process of C/C-SiC composites. Michael C. Halbig [21] pointed out that carbon oxidation takes place in two primary states: the reaction control state and the diffusion control state (referred to as the linear and parabolic states). At lower temperatures, the oxidation corrosion of C/C-SiC composites is predominantly governed by carbon oxidation, whereas at higher temperatures, it shifts to oxidation of the C phase and SiC phase. The specific boundary temperatures also vary under different conditions. The oxidation process of the C phase and SiC phase is influenced by the microstructure of the composites, which is divided into reaction control and diffusion control. It is crucial to clarify the specific control mechanisms to ascertain the path for improvement.

Additionally, there is currently limited research on the oxidation of C/C-SiC composites in high-temperature water vapor environments. Therefore, the main focus of this paper is on exploring the effects of temperature and partial pressure of water vapor on the microstructure and mechanical properties of C/C-SiC composites before and after water vapor corrosion. This includes exploration of the oxidation resistance, structural evolution, and mechanical properties degradation mechanisms of C/C-SiC composites in a high-temperature water vapor environment.

## 2. Experiment

### 2.1. Preparation of C/C-SiC Composites

**Figure 1:**
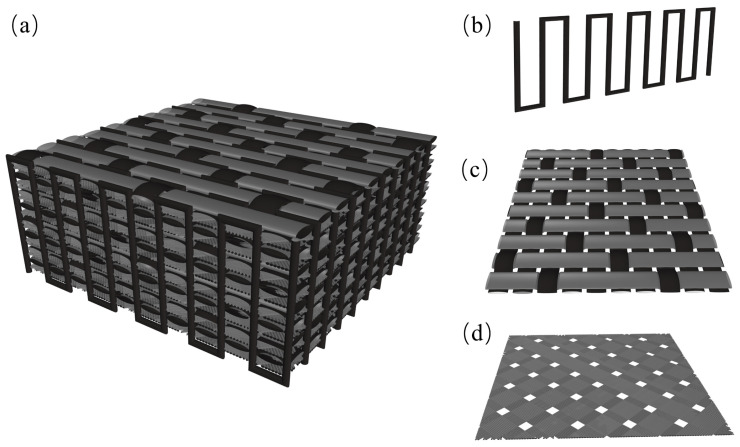
Schematic diagram of stitched prefabricated structure: (**a**) stitched prefabricated body; (**b**) stitched fibers; (**c**) five satin fabrics; (**d**) grid.

A CF preform with a stitched structure was selected for this experiment. The preform was fabricated by repeatedly overlapping a layer of five satin fabrics (HTS, 3 K (these numbers represent the specifications of carbon fiber cloth, where “K” represents the number of carbon fiber filaments contained in a bundle of carbon fiber filaments, and K represents thousands), Toho, Tokyo, Japan) and a layer of ±45° CF mesh (T700, 12 K, Toray, Tokyo, Japan). CFs (HTS, 3 K, Toho, Tokyo, Japan) were used to stitch in the thickness direction with a stitching spacing of 5 mm × 5 mm for single-strand bidirectional stitching (Figure 1a).

PyC was introduced into the CF preform through CVI with a propylene (C_3_H_6_, >99.9% purity, Gaokeqiti, Changsha, China)-nitrogen (N_2_, >99.99% purity, Gaokeqiti, Changsha, China) system. The ratio of propylene to nitrogen flow rate was 3:1 and the sedimentation pressure was maintained between 0.6–0.8 kPa. The porous C/C composites with a density of 1.38 g/cm^3^ were deposited for 150 h at a deposition temperature of 950 °C. Table 1 shows the physical parameters of porous C/C composites.

The C/C-SiC composites were prepared by molten Si penetrating into the porous C/C composites. First, the porous C/C composites were embedded in high-purity Si powder (74 μm, >99.99% purity, Xingrongyuan Technology, Beijing, China) and heated at 1800 °C for 4 h in an Argon (Ar, >99.99% purity, Gaokeqiti, Changsha, China) atmosphere. Then, the melting Si reacted with C above 1414 °C and generated the SiC matrix. Table 1 shows the physical parameters of C/C-SiC composites.

### 2.2. Methods for Water Vapor Corrosion Tests

The experiment involved oxidizing and corroding C/C-SiC composites by mixing water vapor and N_2_ and passing them into a tube furnace. The oxidation behavior of C/C-SiC composites in water vapor was studied by changing the temperature and adjusting the flow rate of water vapor and N_2_ to control the proportion of water vapor content. The main equipment used was a GSL1600X (Kejing, Hefei, China) tube furnace. Before the oxidation test, the tube furnace was opened in the air at 1400 °C for 2 h to remove impurities.

**Figure 2:**
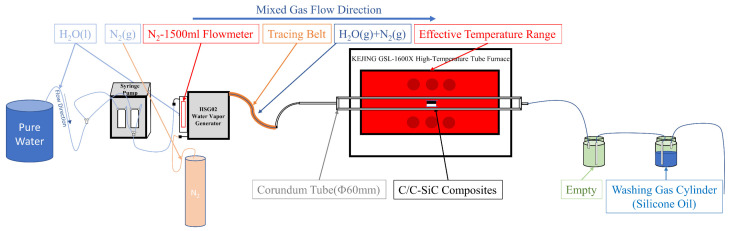
Schematic diagram of experimental equipment.

Firstly, the samples were placed on an alumina crucible which was in the effective temperature zone of the tube furnace. After connecting the washing gas cylinder at the tail, the airtightness of the device was checked by inputting N_2_ into the tube furnace. N_2_ was inputted into the tube furnace at a rate of 500 mL/min to exhaust the air inside the corundum tube and achieve an inert environment. The tube furnace was set to heat up at 10 °C/min to the required temperature for the experiment, and the HSG02 (HXSTAR, Suzhou, China) steam generator and tracing strip were set to heat for 10 min to 300 °C and 160 °C, respectively, until reaching the specified temperature. After the temperature inside the tubular furnace reached the specified temperature, the temperature was held for 2 min to stabilize the temperature inside the furnace. The XFPTS-60D2 (Xunfei Scientific Instrument, Suzhou, China) continuous sampling pump was turned on to input pure water and N_2_ at the set rate. The N_2_ gas was used as a carrier gas to bring saturated water vapor at a controlled temperature into the corundum tube (see Figure 2). The total flow rate of the Ar/H_2_O gas mixture was 1.5 L/min. The time of ventilation was recorded. After reaching the set time, the continuous sampling pump and steam generator were turned off. The cooling program (cooling from the set temperature at a rate of 5 °C/min to 500 °C and then cooling with the furnace, with N_2_ throughout the cooling process to maintain an inert environment) was set for the tube furnace. The samples were taken out for weighing and photographing on the second day.

The oxidation experiments were conducted at different temperatures and in different oxidizing atmospheres. The experimental scheme is shown in Table 2, with a total of 21 oxidation states (Atmosphere 1-21).

### 2.3. Performance Characterization

Three-point bending of the sample before and after oxidation was measured at room temperature by using an electronic universal testing machine (Instron 3369, Instron, Norwood, MA, USA). Six samples were used for each three-point bending test according to the GB/T 40398.2-2021 standard [22] with a span of 40 mm. The size of the test sample was 50 × 10 × 4 mm^3^, and the loading rate was 2.0 mm/min. After that, a scanning electron microscope (SEM, FEI Quanta 250 FEG, FEI, Hillsboro, OR, USA) was used to observe the microstructural changes of C/C-SiC composites before and after corrosion and to observe the fiber extraction after fracture of the composites. The phase compositions of the C/C-SiC composites before and after oxidation were analyzed by X-ray diffraction (XRD, D/max2550 V, Cu Kα1, Rigaku, Tokyo, Japan). The distributions of internal pores in C/C-SiC composites before and after oxidation were observed by computed tomography (CT, Xradia 620 Versa, Carl Zeiss AG, Jena, Germany). The C/C-SiC composite sample was cut and thinned by a dual beam focused ion beam microscope (FIB, Helios Nanolab G3 UC, FEI, Hillsboro, OR, USA). The distribution, thickness, and crystallinity of the oxide layer on the C/C-SiC composites after oxidation were observed by transmission electron microscope (TEM, JEM-F200, JEOL, Tokyo, Japan).

## 3. Results and Discussion

### 3.1. Oxidation Behavior at Different Temperatures

The C/C-SiC composites consist of CF, PyC, SiC, and residual Si. The C phase inside the composites is relatively prone to oxidation. Among them, C fibers begin to be oxidized at 400 °C in an oxygen-containing environment, and single bundles of C fibers above 800 °C can be completely oxidized within a few seconds. In a water vapor environment (without oxygen), CFs and PyC are still oxidized at high temperatures, with gaseous oxidation products CO (or CO_2_) and H_2_, as shown in Equations (1) and (2):

(1) $$C_{\left(s\right)}+{H_{2}O}_{\left(g\right)}={\mathrm{CO}}_{\left(g\right)}+{H_{2}}_{\left(g\right)}$$

(2) $$C_{\left(s\right)}+{2H_{2}O}_{\left(g\right)}={{\mathrm{CO}}_{2}}_{\left(g\right)}+2{H_{2}}_{\left(g\right)}$$

The process of carbon oxidizing in oxygen is exothermal, supplying continuous energy for rapid combustion. The oxidation of carbon when combined with water is akin to a gentle combustion; however, it demands a continuous input of energy to initiate the reaction. The sluggish reaction rate renders it rather selective regarding the oxidative sites, which are typically the defective areas exposed on the carbon surface. The oxidation of carbon with water is strongly reliant on the carbon structures, and the greater the number of defects exposed to the oxidant, the more easily it can be oxidized [20].

Similarly, SiC gradually forms a dense SiO_2_ film in oxygen above 800 °C, which prevents the continued diffusion of oxygen. It was found in previous studies that SiC and Si were oxidized severely in a wet oxygen environment at high temperatures, so the following reactions have to be taken into consideration [11]:

(3) $${\mathrm{SiC}}_{\left(s\right)}+{3H_{2}O}_{\left(g\right)}={{\mathrm{SiO}}_{2}}_{\left(s\right)}+{\mathrm{CO}}_{\left(g\right)}+{{3H}_{2}}_{\left(g\right)}$$

(4) $${\mathrm{Si}}_{\left(s\right)}+{{2H}_{2}O}_{\left(g\right)}={\mathrm{SiO}}_{2(s)}+{2H}_{2(g)}$$

(5) $${{\mathrm{SiO}}_{2}}_{\left(s\right)}+{{2H}_{2}O}_{\left(g\right)\;}={{\mathrm{Si}(\mathrm{OH})}_{4}}_{\left(g\right)}$$

**Figure 3:**
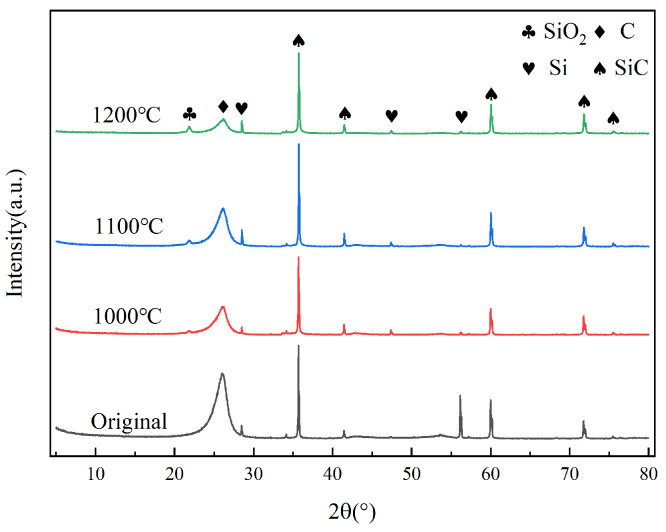
XRD patterns of oxidized samples in original, A3, A12, and A15 states.

The formation of solid SiO_2_ could lead to an increase in the weight of the composites, as shown by Equations (3) and (4), but the oxidation of the carbon phase and the following reaction of SiO_2_ can also lead to a decrease in the weight of the composites, as shown by Equations (1), (2) and (4). The solid product of Equations (1)–(5) is only SiO_2_, which corresponds to the phase composition of the C/C-SiC composite in the four states shown in Figure 3. Si(OH)_4_ is the main product in the reaction between SiO_2_ and water vapor at high water vapor partial pressure and temperature of 1300 °C. The partial pressure of this substance is highly dependent on the partial pressure of water vapor, but it has a small temperature dependence [23].

The diffraction characteristic peaks of C, SiC, and Si are visible in all states, and peaks of SiO_2_ appear after high-temperature oxidation, but the intensity is not high. The crystallinity of SiO_2_ is low, and its relative content is relatively low.

### 3.2. Structural Evolution Before and After Oxidation

**Figure 4:**
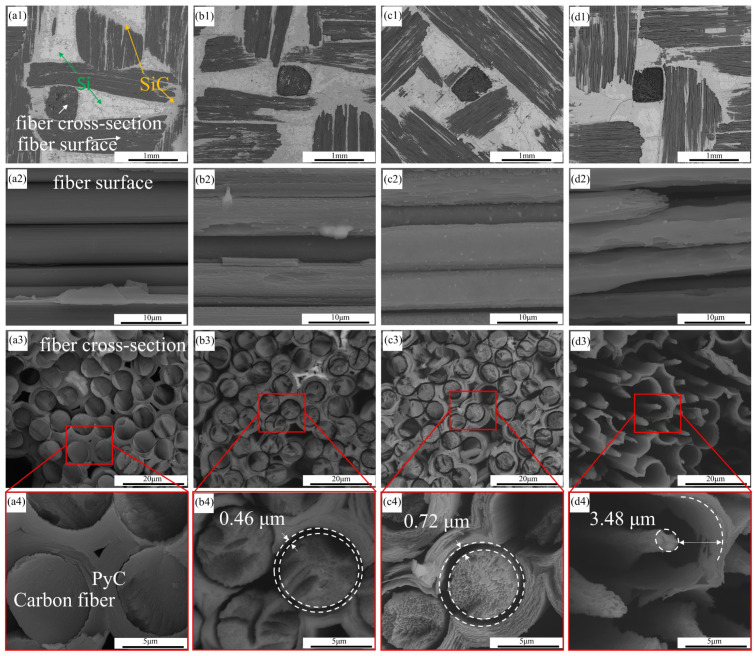
Microstructural changes of C/C-SiC composites after oxidation at different temperatures: (**a1**–**a4**) not corroded; (**b1**–**b4**) A3; (**c1**–**c4**) A12; (**d1**–**d4**) A15.

The microstructural changes of the C/C-SiC composites after oxidation at different temperatures are shown in Figure 4. Firstly, from the SEM images at low magnification (Figure 4(a1,b1,c1,d1)), it can be observed that after high-temperature water vapor oxidation, white stripe-like filaments begin to appear in the CF bundles on the surface of the composites. The lower SiC ceramic phase becomes exposed after the surface CFs are oxidized and consumed. As the oxidation temperature increases, the surface CF bundles become increasingly sparse, and the residual Si with brighter contrast in the ceramic phase also decreases. The residual Si phase in the composites is more susceptible to oxidation than SiC [24].

Secondly, it can be observed (Figure 4(a2,b2,c2,d2)) that the surfaces of the uncorroded CFs are smooth. After corrosion, the surfaces of the CFs become rough; as the oxidation temperature increases, the surfaces of the CFs change from a large number of small pits to a small number of large grooves, and then to a large number of grooves. This phenomenon occurs when the oxidation degree of CFs is relatively low, as oxidation begins at activated points on the CF surfaces. The smooth surface of uncorroded CFs causes oxidation to start from the edge, leading to the formation of numerous small pits. With the appearance of pits, the pit edges become more active and susceptible to oxidation, leading to further oxidation and expansion, forming grooves.

Similarly, the oxidation of the cross-section of the fibers (Figure 4(a3,b3,c3,d3)) also starts from activated points such as edges. It can be observed that the uncorroded CFs are tightly bonded to the matrix with clear boundaries. After corrosion, it can be observed that the original state of tightly wrapped PyC and CFs has changed to a state of detachment. As the oxidation temperature increases, the gap between the CF and PyC layer becomes more obvious. SiC has an average coefficient of thermal expansion of 4.4 × 10^−6^/°C within the temperature range of 25 °C to 1400 °C. CF exhibits negative thermal expansion in its axial direction, but positive thermal expansion in its radial direction. Therefore, the thermal expansion of the fiber and the matrix will reduce the gap generated after oxidation [25,26]. However, due to the small difference in thermal expansion coefficients in this temperature range, the thermal expansion does not affect the trend of being more corroded by water vapor with increasing temperature. Weight loss can also explain this issue. Polyacrylonitrile polymer (PAN) fibers have higher crystallinity inside than at the edges, making them more susceptible to oxidation at the edges. Once oxidized, the fibers’ shape becomes conical [27,28]. As oxidation continues, the surfaces of CFs initially become smooth, followed by the formation of smaller pits. As the oxidation temperature increases, the oxidation phenomenon gradually becomes severe. The activated points on the surfaces are oxidized and disappear, forming larger pits on the surfaces of CFs. The surfaces of CFs change from neat to rough and uneven, and the surrounding PyC layers exhibit a delamination phenomenon.

**Figure 5:**
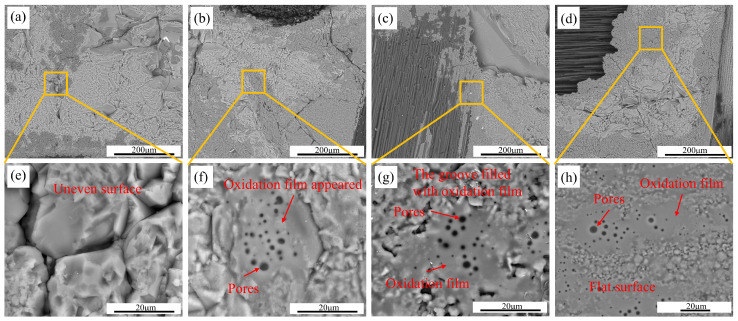
Microstructural changes of C/C-SiC composites after oxidation at different temperatures: (**a**,**e**) not corroded; (**b**,**f**) A3; (**c**,**g**) A12; (**d**,**h**) A15.

Compared to carbon, the changes in SiC and residual Si are less severe, but there is still significant oxidation. Figure 5 shows the SiC matrix on the sample surfaces after oxidation for 60 min under 80% partial pressure of water vapor at different temperatures. It can be observed that after high-temperature water vapor corrosion, there is a thin film on the surface of the SiC matrix. It can be determined that this film is composed of SiO_2_ via EDS surface scanning results. The SiO_2_ film layer prevents further oxidation of the ceramic phase, but the CFs and matrix carbon in C/C-SiC composites are exposed, leading to a fast oxidation rate of carbon. The SiO_2_ layer is extremely thin (see Figure 5) and cannot completely cover the surfaces. Therefore, this layer of oxide film cannot effectively protect the CFs and matrix carbon. As the oxidation temperature increases, the film becomes more pronounced and there are many pores on the surfaces. The reason for this phenomenon may be that as the temperature increases, oxidation products increase, leading to wider film distribution. At temperatures ranging from 1000 to 1200 °C, Si(OH)_4_, formed from SiO_2_, reduces component size. At high temperatures, Si(OH)_4_ escapes in a gaseous state, causing surface bubbles and cracks [29].

**Figure 6:**
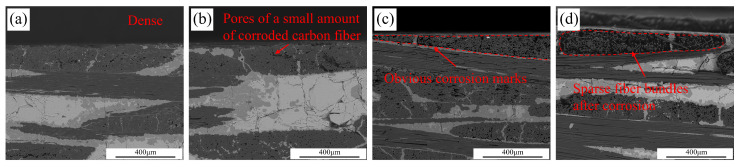
Cross-section corrosion diagram of C/C-SiC composites: (**a**) not corroded; (**b**) A3; (**c**) A12; (**d**) A15.

The cross-sections of the samples after oxidation for 60 min under 80% partial pressure of water vapor at different temperatures are shown in Figure 6. The uncorroded sample has fewer internal pores and tightly arranged fibers. However, after water vapor corrosion, pores begin to appear inside the fiber bundles of C/C-SiC composites. As the temperature increases, the surface fiber bundles become sparser. After water vapor corrosion at 1200 °C, the surface fiber bundles have become very sparse, and the spacing between the fibers becomes larger.

**Figure 7:**
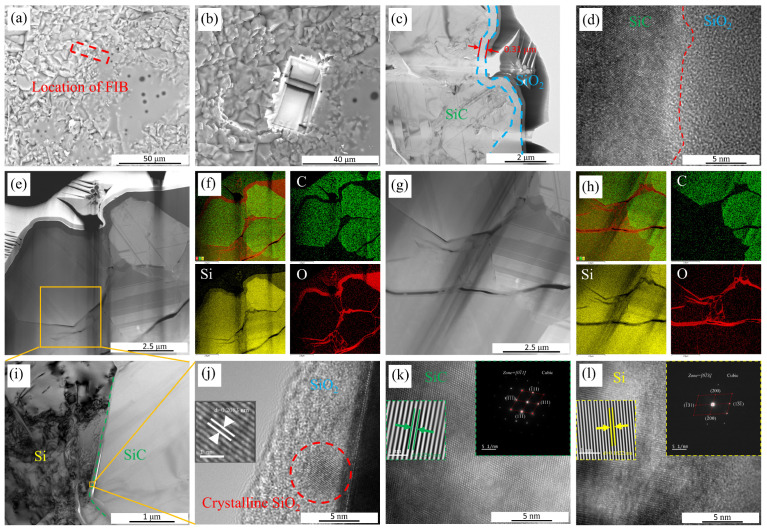
FIB position and TEM schematic diagram of A15-C/C-SiC composites: (**a**) surface of oxidized C/C-SiC composite (before FIB cutting); (**b**) the image shown is the in situ image after FIB cutting; (**c**) HADDF diagram and thickness of SiO_2_ oxide layer; (**d**) HRTEM images of phases a and b; (**e**) HADDF diagram of the surface layer of C/C-SiC composites; (**f**) surface scanning images of C/C-SiC composites; (**g**) HADDF diagram of the inner layer of C/C-SiC composites; (**h**) scanning images of the inner layer of C/C-SiC composites; (**i**) image after adjusting the crystal band axis; (**j**) the crystalline portion within the SiO_2_ oxide layer; (**k**) HRTEM images of SiC; (**l**) HRTEM image of phase Si.

C/C-SiC composites generated SiO_2_ thin films on the surface of the ceramic phase after water vapor oxidation. TEM samples are prepared by FIB processing near the more obvious oxide layer on the surface of the composites. Figure 7 shows the location of FIB and TEM schematic diagrams of C/C-SiC composites after corrosion under the conditions of A15. Figure 7c shows the TEM image of the C/C-SiC composites after corrosion in the A15 state. From the figure, it can be seen that there is a relatively uniform SiO_2_ oxide layer on the surface of the C/C-SiC composite, with a thickness of about 0.31 μm. SiO_2_ presents an amorphous state. The ceramic matrix presents obvious lattice stripes.

Figure 7e–h show the HADDF diagram and its surface scan of the surface and interior of the composite. By scanning the cross-section of the sample, it can be found that SiO_2_ oxide layers exist on both the surface and inside of the sample where cracks exist. It indicates that water vapor fully diffuses into the interior of the sample and reacts with the SiC phase and residual Si phase inside the sample to form an oxide layer. However, SiO_2_ is not completely filling the cracks. The generated SiO_2_ could reduce cracks, leading to a reduction in the channels for water vapor to enter the interior of the composite. In this case, water vapor is partly prevented from entering, and the CFs inside the composite are protected.

Figure 3 reveals the presence of crystalline SiO_2_ within the oxide layer, while Figure 7j reveals the presence of a small portion of crystalline SiO_2_ (in red circle) within the amorphous SiO_2_ oxide layer. The amorphous SiO_2_ is formed by the reaction of SiC with water vapor at 1200 °C. The amorphous SiO_2_ precipitates as a crystalline phase in a high-temperature environment. However, due to the short time at high temperature and the continued reaction of SiO_2_ with water vapor, the content of crystalline SiO_2_ is relatively low. The spacing between the crystalline planes of SiO_2_ is 0.2083 nm.

**Figure 8:**
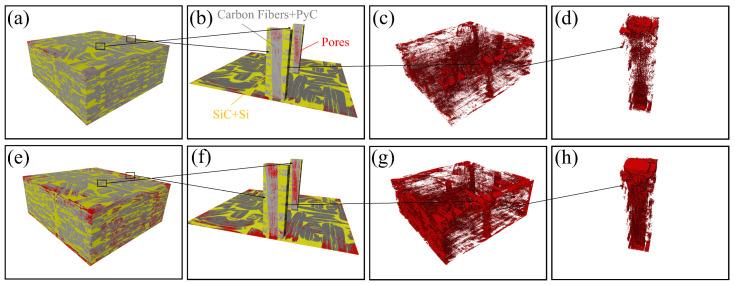
CT images of C/C-SiC composites before and after oxidation: (**a**) overall morphology before oxidation; (**b**) fiber morphology before oxidation; (**c**) pre-oxidation pore distribution; (**d**) pores within the fiber bundle before oxidation; (**e**) overall morphology after oxidation; (**f**) fiber morphology after oxidation; (**g**) pore distribution after oxidation; (**h**) pores in the fiber bundle after oxidation.

Figure 8 shows CT images of C/C-SiC composites before and after oxidation, where the gray phase represents CFs and PyC, the yellow phase represents the ceramic phase (including SiC and residual Si), and the red phase represents pores. After comparing CT images before and after oxidation, it can be seen that the pores significantly increase after oxidation, and mainly gather near the surface CFs and PyC. In comparing (b) and (f), it can be found that the oxidation atmosphere during the oxidation process penetrates the interior of the CF bundles along the pore pathways (including the existing pores in the original CFs and the pores formed during the oxidation process), causing severe oxidation inside the CF bundles.

After high-temperature water vapor oxidation, a comparison of Figure 8c,g shows that the surface of the sample has significantly more pores where it first comes into contact with water vapor. A bundle of stitched fibers was selected from the sample and the inside pores were extracted (Figure 8d,h). It can be clearly seen that the pores on the surface and middle part of the fiber bundle become denser. The interior of the fiber bundle is not directly in contact with water vapor. Instead, the water vapor continues to penetrate the interior of samples through channels formed by water vapor oxidation of surface CFs and PyC. Therefore, it can be preliminarily determined that after oxidation for 60 min in a 1200 °C-80% water vapor environment, the depth of water vapor invasion into the interior of C/C-SiC composites exceeds 2 mm. So, in this state, the damage to CFs is more severe, leading to a significant decrease in mechanical performance.

**Figure 9:**
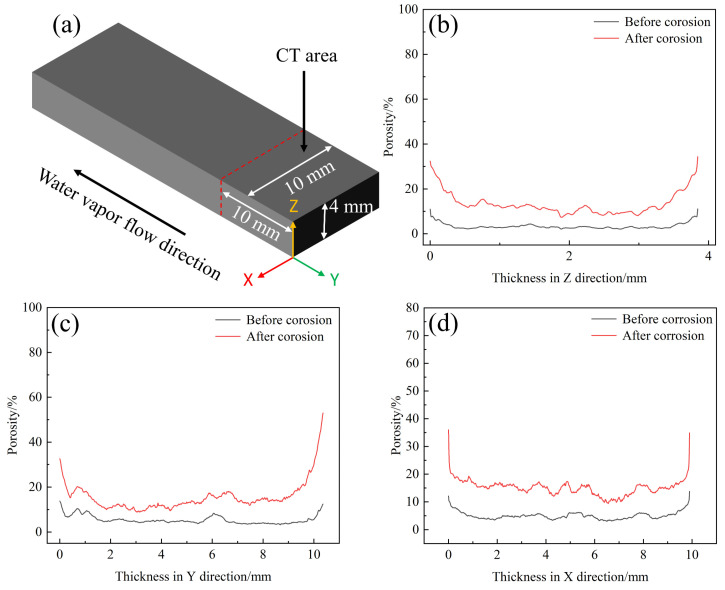
Changes in porosity of C/C-SiC composites before and after corrosion: (**a**) CT area schematic diagram; (**b**) XY surface; (**c**) XZ plane; (**d**) YZ surface.

Figure 9 shows a schematic diagram of the changes in porosity before and after C/C-SiC composites corrosion. It can be seen that the porosity after corrosion is higher than before in all directions. At the edges of all directions, the porosity after corrosion is much higher than before, indicating that all surfaces of the composite in contact with water vapor have severe oxidation. By observing the variation curves of the porosity in the three directions, it can be found that there is a certain pattern in the z-direction (i.e., the porosity in the xy plane). The CFs and ceramic phases inside the composite also alternate with the stacking of carbon cloth layers. After being corroded by water vapor, the internal fibers are oxidized and form pores, while the ceramic phase oxidizes relatively lightly. The ceramic phase oxidizes with solid products, without forming pores, resulting in a periodic distribution of porosity in the composites after oxidation.

### 3.3. Weight Loss Curve

**Figure 10:**
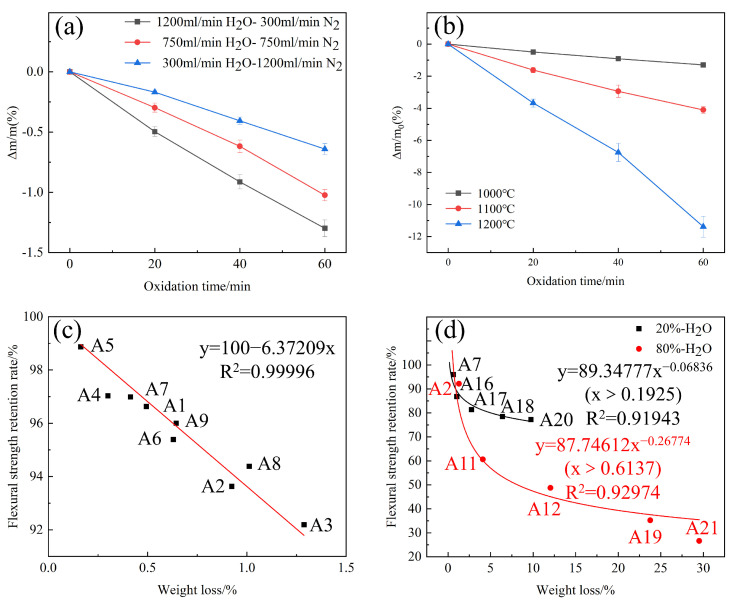
Weight loss curve of C/C-SiC composites: (**a**) the weight change curve of C/C-SiC composites under different partial pressures of water vapor at 1000 °C; (**b**) weight change curve of C/C-SiC composites at 80% partial pressure of water vapor and different temperatures; (**c**) strength retention weight loss relationship curve of C/C SiC composites at 1000 °C; (**d**) strength retention weight loss relationship curve of C/C SiC composites at 1000–1200 °C.

The most straightforward way to observe the samples after oxidation is through weight change. Therefore, we calculated the weight loss during oxidation at different temperatures. Figure 10 shows the weight loss curve of C/C-SiC composites in a high-temperature water vapor environment. The rate of weight loss continuously increases with the increase of oxidation temperature, partial pressure of water vapor, and oxidation time. When the temperature remains constant at 1000 °C, the C/C-SiC composites exhibit a linear weight loss trend as the corrosion time increases at different partial pressures of water vapor. The rate of weight loss (i.e., the slope of the weight loss curve) increases with the increase of partial pressure of water vapor. Similarly, when the partial pressure of water vapor is fixed at 80%, the C/C-SiC composites still exhibit a linear weight loss trend as the oxidation time increases at different temperatures. The rate of weight loss increases with the increase in temperature. At 1200 °C, the rate of weight loss increases significantly compared to 1000 °C and 1100 °C.

Figure 10c,d shows the curve between flexural strength and weight loss rate of C/C-SiC composites. Firstly, Figure 10c plots multiple sets of experimental data at 1000 °C (including different water vapor content and oxidation time) in a coordinate system, and establishes the relationship curve between the two through linear fitting. It can be found that the curve between the flexural strength and weight loss rate of C/C-SiC composites at 1000 °C basically conforms to a linear pattern (R^2^ = 0.99996), with the strength retention rate decreasing as weight loss increases. Figure 10d also plots the experimental data points at 1100 °C and 1200 °C in the coordinate system. After trying to fit multiple curves, the curve between the flexural strength retention rate and weight loss is approximately transformed into a power function. The power function corresponding to different water vapor contents is different (R^2^ values are all greater than 0.9). Under the same weight loss, the group with higher water vapor content exhibits lower flexural strength. This indicates that the decrease in flexural strength retention rate varies with the increase in weight loss rate at different temperature ranges. The connection between flexural strength retention rate and weight loss rate should be divided according to different temperature ranges.

The varying weight loss–strength retention curves under different partial pressures of water vapor may be attributed to different control mechanisms. When the partial pressure of water vapor is low, the reaction rate is mainly controlled by diffusion. When the partial pressure of water vapor is high, the reaction rate is mainly controlled by the reaction. When exposed to high water vapor pressure, the samples experience deeper corrosion, resulting in more severe damage to internal CFs and a lower retention rate of mechanical properties. There is another reason why CFs are severely damaged when the partial pressure of water vapor is high: the surface reaction rate depends on the type of carbon, leading to selective surface reactions. Oxidation is preferably carried out along the fiber axis at the fiber/matrix interface [30]. In addition, more ordered graphite carbon is less sensitive to oxidation [28,30]. The following experiment compares the reaction activity of CFs and PyC by calculating their activation energy in a high-temperature water vapor environment during the oxidation process.

**Figure 11:**
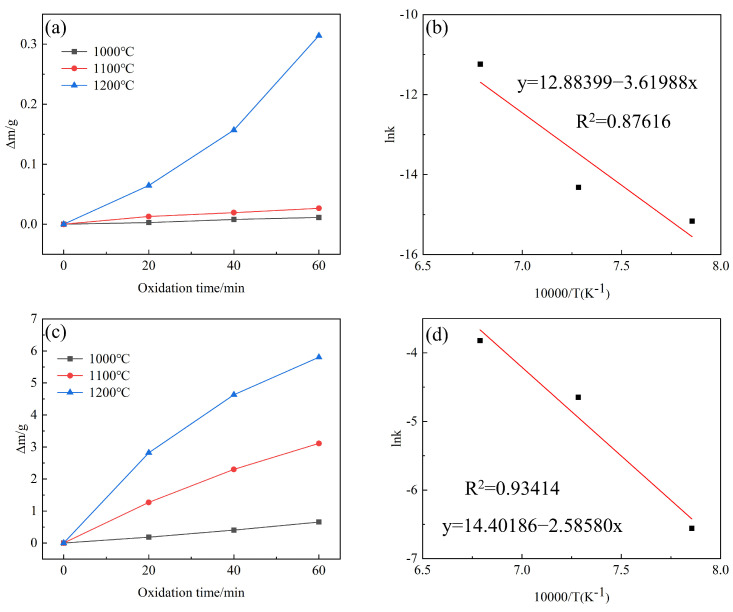
Kinetic curve of C/C-SiC composites oxidation: (**a**) weight loss curves of pyrolysis carbon at different temperatures; (**b**) kinetic curve of pyrolysis carbon oxidation; (**c**) weight loss curves of CFs at different temperatures; (**d**) oxidation kinetics curve of CFs.

A layer of SiC (approximately 40 μm) was deposited on the surface of block graphite. Afterward, PyC was deposited until it could not continue to adhere [31]. The block graphite was oxidized under A3, A12, and A15 to obtain the rate of change in pyrolysis carbon mass (see Figure 11c).

The parameter of mass change is used to describe the reaction rate. Since the rate of mass change of PyC varies linearly with oxidation time, Equation (5) can be used to define the reaction rate constant *k*:

(6) $$(m_{0}-m)/m_{0}=\mathrm{kt}$$

where m_0_, m is the initial mass of PyC before oxidation, and the mass of PyC after oxidation, respectively; *t* is the oxidation time, in minutes; *k* is the reaction rate constant, and the value of *k* is only related to temperature. The rate constant *k* of the PyC oxidation reaction is given by the slope of the weight loss curve in Figure 11c. According to the Arrhenius equation, it has the following relationship with the reaction activation energy [32,33]:

(7) $$\ln k\;=-E_{a}/RT+\mathrm{lnA}$$

Here, *E_a_* is the reaction activation energy, in kJ/mol; R is the gas constant, in J/(mol·K); *T* is the oxidation temperature, in K; A is the pre-exponential factor. After transferring Equation (6) to Equation (7), we can conclude the following:

(8) $$\ln(\left(m_{0}-m\right)/m_{0}t)=-E_{a}/RT+lnA$$

Under static oxidation experimental conditions, for a certain temperature and oxidation time, the (lnA − ln*t*) term is a constant, with $\ln(\left(m_{0}-m\right)/m_{0}t)$ as y and 1/T as x. The kinetic curves of PyC at different oxidation temperatures shown in Figure 11d can be obtained. The activation energy required for the reaction can be calculated from the slope of this curve. The k_PyC_ and k_Cf_ are shown in Table 3.

After calculation, the following can be concluded:

E_aPyC_ = 300.88 kJ/mol

Consistent with the calculation process of PyC, the activation energy of CFs was calculated as follows:

E_aCf_ = 214.98 kJ/mol

As mentioned above, there is a significant difference in the oxidation kinetics between CFs and PyC. The surface reaction rate depends on the structure, microstructure, and chemical composition of carbon (heteroatoms), which can lead to selective surface reactions [24]. Oxidation primarily occurs at the fiber/PyC interface in composites, extending along the fiber axis, resulting in a conical shape near the surface of the CFs during oxidation. The mass loss changes linearly within 1 h of oxidation under water vapor (Figure 10).

In a high-temperature water vapor environment, the water vapor initially reacts with CFs. As the CFs are oxidized, additional diffusion paths can be opened. The continuous reaction on the CF surfaces could consume the water vapor that diffuses into the interior of the samples. Secondly, microcracks commonly occur in C/C-SiC composites due to mismatched thermal expansion between fibers and matrix [34]. Water vapor can enter the composite through these cracks [21]. There is PyC inside the fiber bundle, and its oxidation could also expand the diffusion path of water vapor in C/C-SiC composites. The diffusion and reaction process can be approximately reflected by the internal state of the fiber bundles at various temperatures in Figure 6.

**Figure 12:**
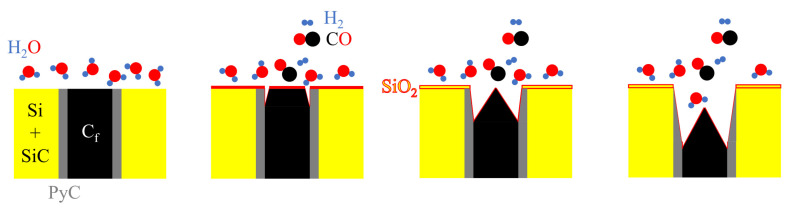
Schematic diagram of water vapor corrosion mechanism of C/C-SiC composites.

As mentioned above, the oxidation law of C/C-SiC composites in high-temperature water vapor is similar to that in oxygen:

(a) CFs, PyC, and SiC are exposed on the surface of C/C-SiC composites.
(b) In a high-temperature water vapor environment, according to its reactivity, CFs are first oxidized. The corrosion of CFs preferentially occurs at the edge, which is the interface between the fiber and the matrix (active points). The corrosion of CF causes debonding and an increase of the reaction area with water vapor (red part of the surface of CFs in the second stage of Figure 12).
(c) CFs continue to be oxidized until the increase of corrosion depth leads to a larger reaction area between PyC and water vapor, exhibiting a similar oxidation rate to CFs. The oxidation continues to occur inside the C/C-SiC composites when a balance is reached between the diffusion and consumption of water vapor. However, as the head of the oxidation area penetrates the interior of the composites, the supply of water vapor could be limited by diffusion, resulting in a decrease in the overall oxidation rate. The oxidation rate of CFs is higher than that of PyC, and the oxidation interface continues to penetrate the interior of the composites along the CF axis.

### 3.4. Three-Point Bending Test

**Figure 13:**
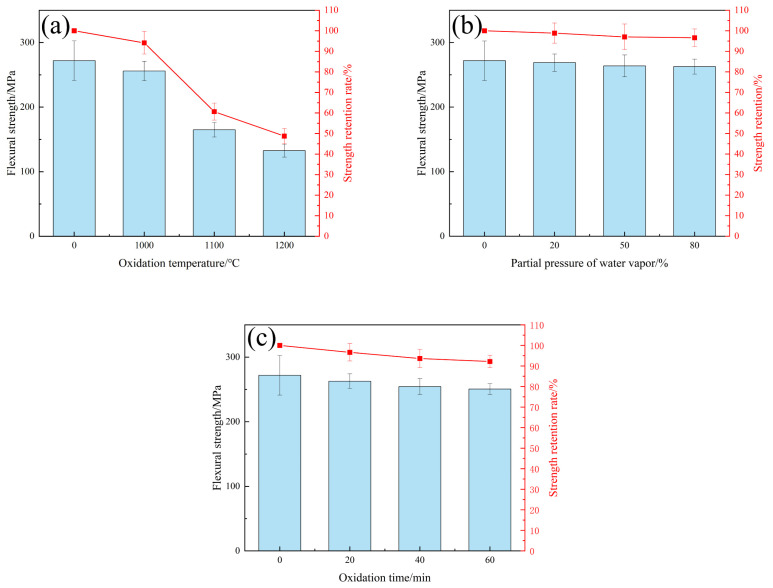
Flexural properties of C/C-SiC composites: (**a**) A3, A12, and A15 flexural strength and strength retention rate; (**b**) A5, A4, and A1 flexural strength and strength retention rate; (**c**) A1, A2, and A3 flexural strength and strength retention rate.

After oxidation, the internal damage of C/C-SiC composites is obvious, which inevitably leads to a decrease in their mechanical properties. The following mainly explores how flexural strength varies with the degree of oxidation. Figure 13 shows the flexural strength results of C/C-SiC composites. As shown in the figure, the average flexural strength of C/C-SiC composites at room temperature is 271.86 ± 30.54 MPa. As the corrosion temperature increases, the flexural strength of the composites gradually decreases. After oxidation at 1200 °C, the flexural strength of the composites sharply decreases, leaving only 132 MPa and a strength retention rate of only 48.75%, less than half. This indicates that the CFs and matrix inside the composites are severely oxidized, leading to a sharp decrease in strength. Si(OH)_4_ formed from SiO_2_ at 1000–1200 °C reduces the size of the components, resulting in surface bubbles and cracks, ultimately leading to degradation of the mechanical properties of the composites [23]. When composites are heated, stress is generated at the interface due to the different thermal expansion coefficients of CFs and matrix. A decrease in interface bonding strength may occur. This problem also occurs during the preparation of composite materials, but the cracks caused by water vapor corrosion at the interface will reduce the impact of this stress. The main reason for the decrease in mechanical properties after high-temperature water vapor corrosion is still the corrosion damage to CFs.

**Figure 14:**
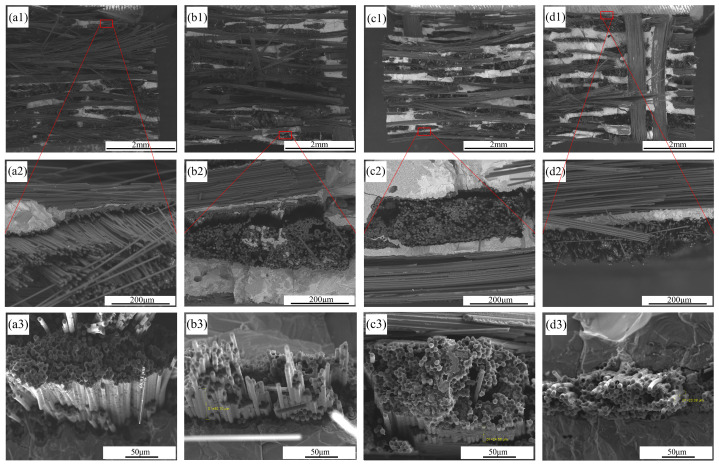
SEM images of mechanical failure of fibers in C/C-SiC composites: (**a1**–**a3**) not corroded; (**b1**–**b3**) A3; (**c1**–**c3**) A12; (**d1**–**d3**) A15.

Figure 14 shows the surface morphology of the flexural fracture of the C/C-SiC composites after oxidation by water vapor under different temperatures. The C/C-SiC composites show numerous fiber bundles pulled out in the central and edge areas, as well as areas where the fibers are flush with the matrix, indicating that C/C-SiC composites exhibit both strong fiber-matrix interface bonding and moderate (or weak) interface bonding. Under strong interface bonding, there could be increased strength for composites, but this cannot exert a toughening effect of fibers. Moderate interface bonding can absorb the energy of partial crack propagation through interface debonding to improve strength. After the interface debonding, there could be relative sliding between the fibers and the matrix. In this case, friction is generated, toughness is improved, and there will be no catastrophic brittle fractures. However, under weak interface bonding, the matrix cannot effectively transmit load, resulting in lower mechanical properties.

After high-temperature water vapor corrosion, there are still a large number of fiber bundles pulled out in the central area of C/C-SiC composites. But the fiber bundles in the edge areas with severe corrosion are mostly flush with the matrix, and the phenomenon of pulling-out is not obvious. The reason for this phenomenon is that CFs react with water vapor, causing damage and thinning of the CFs near the surface. The load-bearing capacity of CFs is therefore lowered. Once cracks extend to the interface of the fiber and matrix, the CFs directly fracture. The CFs cannot achieve an effective toughening effect. The load–displacement curve shows that as the corrosion temperature increases, the deeper the water vapor invades, causing more CFs to be affected. The load–displacement curve gradually transitions from pseudoplastic fracture to brittle fracture [32,35,36].

**Figure 15:**
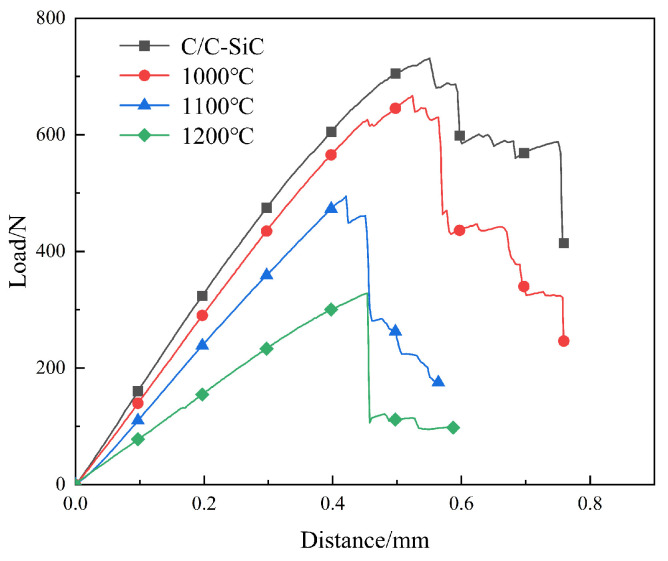
Flexural load–displacement curve.

Figure 15 shows the load–displacement curves of C/C-SiC composites. The flexural performance of C/C-SiC composites is mainly controlled by the inherent strength of continuous fibers and the continuous fiber content along the load direction. After oxidation by water vapor, the flexural strength of C/C-SiC composites decreased, but the samples still maintained pseudoplastic fracture characteristics at different temperatures. The ascending stage is divided into linear deformation and nonlinear deformation which exceeds the elastic limit. After reaching the maximum load, it descends in a stepped manner. As the temperature increases, the amplitude of the stepped fluctuation increases. This indicates that there is some fiber damage near the surface of the C/C-SiC composites after oxidation by water vapor, resulting in a certain degree of decrease in the flexural strength of the composites. As the load continues to increase, the number of cracks continues to increase, and the fibers are pulled out and broken. The load–displacement curve rapidly decreases until the crack extends to the next layer of fiber cloth, repeating crack deflection, etc. Therefore, the load–displacement curve shows a stepped decrease.

## 4. Conclusions

The difficulty of oxidation in water vapor can be ranked from easy to difficult in the following order: CF, PyC, and ceramic phase. The SiO_2_ layer on C/C-SiC composites could not provide effective protection in a high-temperature water vapor environment. After high-temperature water vapor corrosion, the mechanical performance decreased, with the extent of decline increasing as oxidation temperature, oxidation time, and partial pressure of water vapor increased. The fracture mode transitioned from “pseudoplastic” fracture before corrosion to brittle fracture after corrosion. When the water vapor content is low, the oxidation rate is controlled by diffusion, and the oxidation rates of CF and PyC are similar. When the water vapor content is high, the oxidation rate is controlled by the reaction, and CFs are more corroded, resulting in a significant decrease in the strength retention rate. Based on the above research, we believe it is worth further studying the oxidation kinetics details of CF and PyC under different water vapor contents, including determining the specific reaction rate equations and activation energy parameters for diffusion control and reaction control stages, as well as studying how to adjust the oxidation rate under the two control mechanisms by adding specific elements or changing the process to improve the overall water vapor oxidation resistance of the composite.

## Figures and Tables

**Table 1:** Physical parameters of composites.

Composite	Prefabricated Density (g·cm^−3^)	Density (g·cm^−3^)	Matrix	Fiber Volume Fraction (%)	Porosity (%)
C/C	0.83	1.38	PyC	46.11	21.95
C/C-SiC	-	2.05	PyC + SiC	-	5.43

**Table 2:** Water vapor corrosion parameters of C/C-SiC composites.

State	Temperature (°C)	Flow Rate of Water Vapor (mL/min)	Flow Rate of N_2_ (mL/min)	Partial Pressure of Water Vapor (%)	Oxidation Time (min)
A1	1000	1200	300	80	20
A2	1000	1200	300	80	40
A3	1000	1200	300	80	60
A4	1000	750	750	50	20
A5	1000	300	1200	20	20
A6	1000	750	750	50	40
A7	1000	300	1200	20	40
A8	1000	750	750	50	60
A9	1000	300	1200	20	60
A10	1100	1200	300	80	20
A11	1100	1200	300	80	40
A12	1100	1200	300	80	60
A13	1200	1200	300	80	20
A14	1200	1200	300	80	40
A15	1200	1200	300	80	60
A16	1100	300	1200	20	40
A17	1200	300	1200	20	40
A18	1300	300	1200	20	40
A19	1300	1200	300	80	40
A20	1400	300	1200	20	40
A21	1400	1200	300	80	40

**Table 3:** Oxidation rate of PyC/C_f_.

Temperature (°C)	k_PyC_ (10^−7^·min^−1^)	k_Cf_ (min^−1^)
1000	2.59751	0.00142
1100	6.04556	0.00958
1200	131.191	0.02190

## Data Availability

The original contributions presented in this study are included in the article. Further inquiries can be directed to the corresponding author.
